# Argonaute5 and its associated small RNAs modulate the transcriptional response during the rhizobia-*Phaseolus vulgaris* symbiosis

**DOI:** 10.3389/fpls.2022.1034419

**Published:** 2022-11-17

**Authors:** María del Socorro Sánchez-Correa, Mariel C. Isidra-Arellano, Eithan A. Pozas-Rodríguez, María del Rocío Reyero-Saavedra, Alfredo Morales-Salazar, Sarah Melissa Lugo-Caro del Castillo, Alejandro Sanchez-Flores, Verónica Jiménez-Jacinto, Jose L. Reyes, Damien Formey, Oswaldo Valdés-López

**Affiliations:** ^1^ Laboratorio de Genómica Funcional de Leguminosas, Facultad de Estudios Superiores Iztacala, Universidad Nacional Autónoma de México, Tlalnepantla, Estado de México, Mexico; ^2^ Centro de Ciencias Genómicas, Universidad Nacional Autónoma de México, Cuernavaca, Morelos, Mexico; ^3^ Unidad Universitaria de Secuenciación Masiva y Bioinformática, Instituto de Biotecnología, Universidad Nacional Autónoma de México, Cuernavaca, Morelos, Mexico; ^4^ Departamento de Biología Molecular de Plantas, Instituto de Biotecnología, Universidad Nacional Autónoma de México, Cuernavaca, Morelos, Mexico

**Keywords:** argonaute proteins, microRNAs, rhizobial tRNA-derived sRNA fragments, root nodule symbiosis, legumes

## Abstract

Both plant- and rhizobia-derived small RNAs play an essential role in regulating the root nodule symbiosis in legumes. Small RNAs, in association with Argonaute proteins, tune the expression of genes participating in nodule development and rhizobial infection. However, the role of Argonaute proteins in this symbiosis has been overlooked. In this study, we provide transcriptional evidence showing that Argonaute5 (AGO5) is a determinant genetic component in the root nodule symbiosis in *Phaseolus vulgaris*. A spatio-temporal transcriptional analysis revealed that the promoter of *PvAGO5* is active in lateral root primordia, root hairs from rhizobia-inoculated roots, nodule primordia, and mature nodules. Transcriptional analysis by RNA sequencing revealed that gene silencing of *PvAGO5* affected the expression of genes involved in the biosynthesis of the cell wall and phytohormones participating in the rhizobial infection process and nodule development. PvAGO5 immunoprecipitation coupled to small RNA sequencing revealed the small RNAs bound to PvAGO5 during the root nodule symbiosis. Identification of small RNAs associated to PvAGO5 revealed miRNAs previously known to participate in this symbiotic process, further supporting a role for AGO5 in this process. Overall, the data presented shed light on the roles that PvAGO5 plays during the root nodule symbiosis in *P. vulgaris*.

## Introduction

Legumes can fulfill their nitrogen needs by forming endosymbiosis with nitrogen-fixing soil bacteria collectively known as rhizobia. This symbiosis involves the modification of lateral roots to form the so-called root nodules (Schiessl et al., 2019; Soyano et al., 2019). These organs provide the appropriate environment for rhizobial nitrogenase to convert atmospheric nitrogen into ammonium. The formation of the root nodules requires the simultaneous and coordinated activation of two genetic programs. These programs allow the reactivation of cell division of the root cortex to form the nodule meristem and the infection of nodule cells by the rhizobia (Roy et al., 2020).

Legumes and rhizobia must communicate through diffusible signal molecules to activate the signaling networks required to establish the root nodule symbiosis. Legumes release flavonoids to the rhizosphere, where compatible rhizobia detect them (Phillips et al., 1992; Liu and Murray, 2016). In response, rhizobia release lipochitooligosaccharides (LCOs) that the legume host perceives through a set of plasma-membrane-located LysM domain receptor kinases (Dénarié et al., 1996; Broghammer et al., 2012; Murakami et al., 2018). Upon sensing these rhizobia-derived LCOs, a series of molecular responses are activated (Roy et al., 2020). Among them are rapid and continuous oscillations in nuclear and perinuclear calcium concentrations (Ehrhardt et al., 1996; Kosuta et al., 2008). Calcium-Calmodulin Kinase further decodes these calcium signatures, subsequently phosphorylating the transcription factor (TF) CYCLOPS (Lévy et al., 2004; Singh and Parniske, 2012). CYCLOPS, through the action of DELLA proteins, forms a large complex with the TFs Nodule Signaling Pathway 2 (NSP2) and NSP1 to activate the expression of the *Nodule INception* (*NIN*) TF gene (Oldroyd and Long, 2003; Hirsch et al., 2009; Cerri et al., 2012; Jin et al., 2016). NIN, in turn, controls the expression of genes participating in the rhizobial infection, nodule development, and regulation of nodule number per root (Hirsch et al., 2009; Cerri et al., 2012; Soyano et al., 2014; Jin et al., 2016).

The root nodule symbiosis is also finely tuned by diverse small RNA (sRNA) classes, with microRNAs (miRNAs) being the most studied (Tiwari et al., 2021). miRNAs are approximately 21-nt long and control gene expression by mRNA cleavage, translational inhibition, or DNA methylation (Wang et al., 2019). miRNAs have a determinant role in regulating nodule development and, to some extent, the colonization of root nodule cells by rhizobia (Tiwari et al., 2021). For instance, miR169 targets the *Nuclear Factor-YA1* gene (formerly called *HAP2*), which controls nodule meristem persistence and the progression of rhizobial infection in *Medicago truncatula* (Combier et al., 2006). miR171c plays a role during the rhizobial infection stage by targeting the *NSP2* TF gene in *Lotus japonicus* (De Luis et al., 2012). Evidence in *L. japonicus* and *M. truncatula* indicates that miR2111 is a mobile miRNA required to trigger the nodule development program (Tsikou et al., 2018; Okuma et al., 2020).

Modulating the legume host defense is also crucial for a successful symbiosis with rhizobia (Berrabah et al., 2015; Wang et al., 2016; Berrabah et al., 2019). Evidence in *Glycine max* and *M. truncatula* indicates that miR482, miR2109, and miR2118, which target *NB-LRR* genes – encoding receptors that recognize specific pathogen effectors and trigger plant resistance responses – modulate the legume immune response during the root nodule symbiosis (Li et al., 2010; Sós-Hegedus et al., 2020). This symbiosis is not only regulated by legume-derived sRNAs. Recently, it was reported that rhizobial transfer RNA (tRNA)-derived sRNA fragments (tRFs) help regulate the expression of host genes participating in the rhizobial infection and nodule development in *G. max* (Ren et al., 2019).

ARGONAUTE proteins (AGOs) are present in eukaryotes and associate with sRNAs to form the RNA-induced silencing complex (RISC) which regulates the expression of sRNA target genes (Hutvagner and Simard, 2008). Eukaryotic AGOs are structurally conserved and contain four domains: a variable N-terminal domain, PIWI-ARGONAUTE-ZWILLE (PAZ), MIDdle (MID), and PIWI (Tolia and Joshua-Tor, 2007). The PAZ domain binds sRNAs, while the MID domain recognizes the 5’ nucleotide of sRNAs. The PIWI domain adopts an RNase H-like fold, allowing most AGOs to cleave target messenger RNAs complementary to the bound sRNAs (Song et al., 2004). The number of AGO genes present in plant genomes is variable and is plant species-dependent. For instance, the *Arabidopsis thaliana* genome encodes 10 AGOs (Vaucheret, 2008). In contrast, there are 17 in maize (Qian et al., 2011), 19 in rice (Kapoor et al., 2008), and 14 in *Phaseolus vulgaris* (Reyero-Saavedra et al., 2017).

In plants, both sRNAs and AGOs tune diverse developmental processes and coordinate adaptation to the environment by serving as sequence-specific regulators of genes (Manavella et al., 2019). For instance, the expression of seventeen maize AGOs is differentially regulated in response to cold, salinity, drought, and abscisic acid addition, suggesting that AGOs are determinant in the adaptation to these stresses (Zhai et al., 2019). The participation of diverse sRNAs during nodule development suggests that AGOs also play roles in the root nodule symbiosis. Indeed, we previously reported that AGO5 expression increases in response to rhizobia in both *P. vulgaris* and *G. max* and demonstrated that silencing *AGO5* reduces both nodule size and the number of rhizobia-infected nodule cells (Reyero-Saavedra et al., 2017). We therefore hypothesize that AGO5 plays roles in coordinating the genetic programs involved in rhizobial infection and nodule development.

In this study, we provide new evidence demonstrating that AGO5 contributes to regulation of the rhizobial infection process and nodule development in *P. vulgaris*. A spatio-temporal analysis in *P. vulgaris promoterPvAGO5:GUS (pPvAGO5:GUS)* transgenic roots revealed that *AGO5* is expressed in root hairs in response to rhizobia. This analysis also indicates that the *PvAGO5* promoter has strong activity in nodule primordia and mature nodules. Further transcriptional analysis by genome-wide mRNA sequencing revealed that gene silencing of *PvAGO5* affects the expression of key nodulation genes as well as others related to the biosynthesis of the cell wall and phytohormones involved in the rhizobial infection process and nodule development. PvAGO5 immunoprecipitation coupled with sRNA sequencing revealed the sRNAs bound into AGO5 during the root nodule symbiosis. Among the sRNA associated to AGO5 were members of miR166, miR319, miR396, and miR2118 miRNA families, all of which were previously shown to participate in the root nodule symbiosis in different legumes. We also observed that AGO5’s sRNA cargo in mature nodules contains rhizobial-derived tRFs that target *P. vulgaris* genes also implicated in this symbiosis. The data presented shed new light on AGO5’s participation during the root nodule symbiosis in *P. vulgaris*.

## Material and methods

### Plant materials

*P. vulgaris* cultivar Negro Jamapa was used in this study. Seeds were surface sterilized and germinated as reported in Reyero-Saavedra et al. (2017). Two-day-old seedlings were transferred to 2 L pots containing moist perlite, kept in a growth chamber at 25-27°C, and watered with Summerfield nutrient solution (Summerfield et al., 1977) every three days.

### Bacterial strains and culture conditions

*Rhizobium tropici* CIAT 899 strain was used to inoculate *P. vulgaris* seedlings. *R. tropici* cells were grown for two days at 30°C on PY medium (5 g/L peptone; 3 g/L yeast extract) supplemented with 0.7 M CaCl_2_ and 20 μg/mL nalidixic acid. After two days, *R. tropici* cells were harvested and resuspended in sterile water at O.D_600nm_= 0.3. One mL of this bacterial suspension was used to inoculate *P. vulgaris* seedlings individually.

Empty binary vectors pKGWFS7 and pTDT-DC-RNAi were propagated in *Escherichia coli* DB3.1, *promoterPvAGO5::GUS::GFP*, and the RNA interference (RNAi) against *PvAGO5* (see below for details of these genetic constructs) were propagated in DH5α.

*Agrobacterium rhizogenes* K599 strain was used to generate transgenic roots in *P. vulgaris* plants (see below for details). *A. rhizogenes* cells were grown on Luria-Bertani (LB) plates for two days at 30°C. 100 μg/mL spectinomycin was added to select for the presence of plasmid vectors.

### Plasmid construction

To analyze *PvAGO5* promoter activity, a 1,800 bp DNA fragment upstream of the start codon was PCR-amplified from genomic DNA of *P. vulgaris* var. Negro Jamapa using specific primers. The amplified fragment was then cloned into the pENTR-D-TOPO (Thermo Fisher Scientific) vector. The resulting pENTR-*pPvAGO5* plasmid was recombined into the pKGWFS7 binary vector containing *GUS* and *GFP* CDS, yielding the transcriptional *pPvAGO5::GUS::GFP* fusion.

A previously generated and reported RNAi construct was used to silence the expression of *PvAGO5* (Reyero-Saavedra et al., 2017).

All constructs were verified by DNA sequencing. Primer sequences for plasmid constructions are shown in **Supplementary Table 1**.

### *Agrobacterium rhizogenes*-mediated transformation

Binary vectors with *pPvAGO5:GUS-GFP* or *PvAGO5*-RNAi constructs were mobilized into *A. rhizogenes* K599 strain by electroporation. The empty vectors pKGWFS7 or pTDT-DC-RNAi were used as controls. *A. rhizogenes*-mediated transformation was performed according to Estrada-Navarrete et al. (2007). *P. vulgaris* composite plants (plants with the transformed root system and untransformed shoot system) were grown in 2 L pots containing wet perlite. Tandem Double Tomato (TDT) or GFP fluorescence in transgenic roots was observed with a fluorescence stereomicroscope.

### Lateral root phenotype under non-symbiotic conditions

To evaluate the effect of the gene silencing of *PvAGO5* in the lateral root development under non-symbiotic conditions, *A. rhizogenes*-mediated *pPvAGO5*-RNAi transgenic roots were generated in the *P. vulgaris* as described above. Transgenic roots expressing an empty vector were used as a control. Composite plants were watered with 5 mM KNO_3_-supplemented Summerfield nutrient solution. After three weeks, transgenic roots showing TDT fluorescence were collected to evaluate the number, density and length of lateral roots. Lateral root density was calculated by dividing the number of lateral roots on each transgenic root with the length of the root. For these experiments, ten biological replicates, each one containing ten transgenic roots from independent composite plants, were included.

### Nodulation assays in common bean wild type and composite plants

Two-day-old *P. vulgaris* wild-type or composite plants expressing the empty vector or the *PvAGO5*-RNAi construct were transferred to 2 L pots containing wet perlite and inoculated with 1 mL of *R. tropici* (O.D._600nm_= 0.3). Plants were watered with nitrogen-free Summerfield nutrient solution (Summerfield et al., 1977) every three days. Inoculated plants were kept in a growth chamber at 25-27°C. Ten and twenty days after rhizobial inoculation, roots with nodule primordia and nitrogen-fixing nodules (mature nodules) were collected separately and immediately processed for PvAGO5 Immunoprecipitation assays or RNA-seq analyses (see below for details). For these experiments, three biological replicates containing six independent wild-type or composite plants were included.

### *AGO5* promoter activity under non-symbiotic conditions

To evaluate *PvAGO5* promoter activity under non-symbiotic conditions, *A. rhizogenes*-mediated *pPvAGO5:GUS-GFP* transgenic roots were generated in the *P. vulgaris* as described above. Composite plants were watered with 5 mM KNO_3_-supplemented Summerfield nutrient solution. After three weeks, transgenic roots showing GFP fluorescence were collected for GUS staining as reported in (Isidra-Arellano et al., 2020). For this experiment, ten biological replicates containing ten composite plants and ten roots were included.

### *AGO5* promoter activity during the root nodule symbiosis

To evaluate *PvAGO5* promoter activity during the root nodule symbiosis, *A. rhizogenes*-mediated *pPvAGO5:GUS-GFP* transgenic roots were generated in the *P. vulgaris*. Composite plants were inoculated with 1 mL of *R. tropici* (O.D._600nm_= 0.3). Rhizobia-inoculated plants were watered with nitrogen-free Summerfield nutrient solution. Upon one, ten and twenty days after rhizobia inoculation, transgenic roots, roots with nodule primordia and mature nodules were collected for GUS staining assays as reported in (Isidra-Arellano et al., 2020). For this experiment, ten biological replicates containing ten composite plants and ten roots were included.

### RNA extraction and RT-qPCR analysis

To analyze the expression of genes listed in **Supplementary Table 1**, transgenic roots and mature nodules expressing the *PvAGO5*-RNAi construct and showing TDT fluorescence were immediately harvested in liquid nitrogen and stored at -80°C until used. Total RNA was isolated from roots and nodules from three different composite plants using the ZR Plant RNA miniprep (Zymo Research, USA) following the manufacturer’s instructions. cDNA was synthesized from 1 μg of genomic DNA-free total RNA and used to analyze gene expression by RT-qPCR as we previously described in Isidra-Arellano et al. (2020). RT-qPCR primer sequences used in this study are provided in **Supplementary Table 1**. Three biological replicates, each one with six technical replicates, were included for this experiment.

### Preparation of messenger RNA-Seq libraries and next-generation sequencing

Total RNA was isolated as described in the previous section from 0.5 g of one-day rhizobia- or mock-inoculated transgenic roots and transgenic mature nodules expressing an empty vector, or the *PvAGO5*-RNAi construct. Stranded messenger RNA-seq (mRNA-seq) libraries were generated from 1 μg of gDNA-free total RNA from each experimental condition and prepared using the TruSeq RNA Sample Prep kit (Illumina, San Diego, CA, USA) according to the manufacturer’s instructions. For each experimental condition, three biological replicates containing six independent composite plants were included. Eighteen libraries were sequenced on an Illumina NextSeq 500 platform with a 150-cycle sequencing kit and a configuration of pair-end reads with a 75 bp read length. Library construction and sequencing were performed by the Unidad Universitaria de Secuenciación Masiva y Bioinformática (Instituto de Biotecnología, UNAM, México).

### Mapping and processing messenger RNA-Seq reads

Adapter and contamination removal were carried out using in-house Perl scripts. Sequences were filtered based on quality (Q33, FASTQ Quality Filter v0.0.13, http://hannonlab.cshl.edu/fastx_toolkit/index.html). About ten million reads per sample were aligned to the *P. vulgaris* transcriptome (v2.1 from Phytozome v13) using Bowtie2 (v2.3.5) and the recommended parameters to match RSEM analysis input requirements (Langmead and Salzberg, 2012). Gene expression was calculated using the RNA-seq by Expectation Maximization (RSEM) method (v1.3.3) and the default parameters (Li and Dewey, 2011). Significantly Differentially Expressed Genes (DEGs: adjusted p-value ≤ 0.05) were identified using DESeq2, part of the Integrated Differential Expression Analysis MultiEXperiment (IDEAMEX) platform (Jiménez-Jacinto et al., 2019), with the RSEM expected counts. Gene Ontology (GO) term enrichment analysis was performed using AgriGO (v2.0) and default parameters (FDR cutoff = 0.05) (Tian et al., 2017). Protein domain enrichment analysis was performed using PhytoMine tool from Phytozome (v13), with Holm-Bonferroni correction (FDR cutoff = 0.05). Heatmaps were created with ggplot2 and heatmap.2 libraries using R software (v4.1.2).

### AGO5 immunoprecipitation, small RNAs isolation and sequencing

Uninoculated roots, roots bearing nodule primordia, and mature nodules from wild-type *P. vulgaris* were manually collected on ice. For each experimental condition, material from 100 plants was ground on ice with immunoprecipitation buffer (50 mM Tris-HCl pH7.5; 1.5 mM NaCl, 0.1% Nonidet P40, 4 mM MgCl_2_, 2 mM DTT, and Sigma Protease inhibitor cocktail). Cell debris was removed by centrifugation twice for 15 min at 12,000 g at 4°C. Next, supernatants were precleared by incubation for 1 hour with 10 μl Protein A-Agarose (Roche). Samples were centrifuged at 1,500 g for 5 minutes at 4°C. Supernatants were incubated with Protein A-Agarose (Roche) supplemented with 2 μl anti-AGO5 for exactly 16 hours with rotation at 4°C. Samples were centrifuged at 1,500 g for 5 minutes at 4°C. The beads were washed three times with a washing buffer (50 mM Tris-HCl pH7.5, 150 mM NaCl, 0.1% Nonidet P40, 4 mM MgCl_2_, 2 mM DTT, and Sigma Protease inhibitor cocktail). Next, beads were resuspended in 0.4 M NaCl and sRNAs were extracted by phenol:chloroform:iso-amyl alcohol extraction.

Nine small libraries (3 for uninoculated roots, 3 for roots bearing nodule primordia, and 3 for mature nodules) were generated from AGO5-bound sRNA using TruSeq small RNA Library Prep kit (Illumina, San Diego, CA, USA) according to the manufacturer’s instructions. Libraries were sequenced on an Illumina NextSeq 500. Library construction and sequencing were performed by the Unidad Universitaria de Secuenciación Masiva y Bioinformática (Instituto de Biotecnología, UNAM, México).

### Small RNA data analysis

Adapters and reads with quality mean lower than 33 were removed and the sequence redundancy collapsed using the FASTX-Toolkit suite (v0.0.13, http://hannonlab.cshl.edu/fastx_toolkit/index.html). Small RNAs were compared to Viridiplantae miRNAs from miRbase (v22), 277 P*. vulgaris* small RNAs previously identified and published in (Formey et al., 2015; Formey et al., 2016), and 10 tRFs from *Rhizobium etli* (Ren et al., 2019). Normalization was performed using DESeq2. Only the sRNAs lying within the top 1% of the most accumulated sequences in a given library, were selected for further analyses. Transcript targets for the identified sRNAs were predicted using psRNAtarget (V2) and default parameters (Dai et al., 2018). Venn diagrams were designed using DeepVenn (Hulsen et al., 2008).

### Statistical analyses and graphics

Statistical analyses and graphic generation were conducted using R software 4.1.2. The specific tests performed are indicated in the legend of the corresponding figure.

## Results

### Down-regulation of *PvAGO5* increases the number and density of lateral roots under non-symbiotic conditions

**Figure 1:**
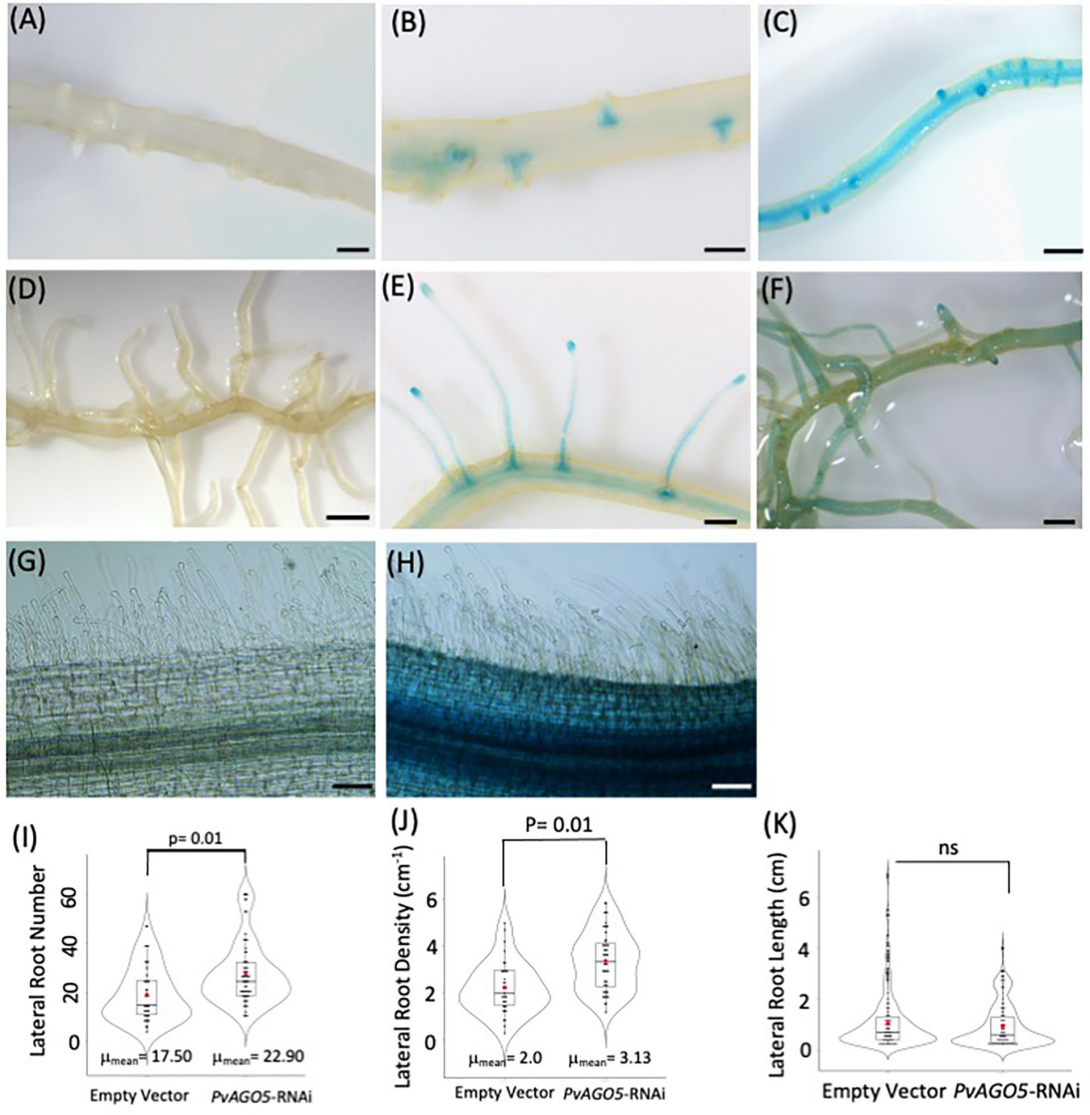
*PvAGO5* is expressed in lateral roots under nonsymbiotic condition. *P. vulgaris composite* plants with transgenic roots expressing either the empty vector **(A, D, G)** or the *pPvAGO5:GUS-GFP* construct **(B, C, E, F, H)** were grown under optimal-nitrogen conditions for three weeks. Roots expressing the *pPvAGO5:GUS-GFP* displayed GUS signal in the lateral roots primordium and mature lateral roots **(B, C, E, F)**. Scale bars represent 500 μm. Images shown are representative of ten biological replicates, each one containing ten transgenic roots. Transgenic roots expressing the empty vector or *pPvAGO5:GUS-GFP* construct shown no GUS activity at the root hairs **(G, H)**. **(I)** Number and density **(J)** of lateral roots formed in transgenic roots expressing the empty vector or the *PvAGO5*-RNAi construct. **(K)** Lateral root length of *PvAGO5*-RNAi transgenic roots compared with the empty vector transgenic roots. Boxes indicate the second and third quartiles, with median and mean values indicated as a line and red dot, respectively. Data shown was obtained from ten biological replicates, each one containing ten transgenic roots. Statistical significance was obtained using Welch’s *t*-test. ns, not significant.

Our previous transcriptional analyses by RT-qPCR showed that *PvAGO5* is preferentially expressed in *P. vulgaris* roots, compared to leaves (Reyero-Saavedra et al., 2017). However, these data do not provide spatiotemporal insights into *PvAGO5* expression in this essential organ for root nodule symbiosis. To tackle this, we cloned a 1.8 kb fragment of the *PvAGO5* promoter (*pPvAGO5*) and generated a transcriptional fusion to the *GUS* (β-glucuronidase) and *GFP* coding sequence. The Empty vector:*GUS-GFP* (control) or the *pPvAGO5:GUS-GFP* constructs were transfected separately into *P. vulgaris* using *A. rhizogenes*-mediated transformation (Estrada-Navarrete et al., 2007), and composite plants were grown and watered with nitrogen-containing Summerfield nutrient solution for three weeks. We observed no GUS activity in transgenic roots expressing the empty vector (**Figures 1A, D**). In contrast, in the absence of rhizobia, 65 out of 70 roots transformed with *pPvAGO5:GUS-GFP* displayed a weak GUS signal in the whole root, intensified to a strong signal in lateral root primordia and mature lateral roots (**Figures 1B, E**); whereas the other five roots showed the same GUS signal intensity through the entire root, (**Figures 1C, F**). GUS activity was absent in the root hairs of the 70 transgenic roots analyzed (**Figures 1G, H**).

The presence of *PvAGO5* activity in the lateral root primordia prompted us to compare the number, density, and length of lateral roots in *P. vulgaris* composite plants expressing an empty vector (control) or the *PvAGO5*-RNAi construct. This analysis indicated that the number of lateral roots and density was increased 15% in *PvAGO5*-RNAi transgenic roots compared to the empty vector controls, with no significant differences in root length (**Figures 1I–K**). Moreover, no growth defects in the root hairs were observed in *PvAGO5*-RNAi transgenic roots (**Supplementary Figure 1**). In summary, these data inform that *PvAGO5* is expressed mostly in lateral root primordia in the absence of rhizobia. Furthermore, they also suggest that AGO5 plays a role in lateral root development in *P. vulgaris*.

### *PvAGO5* is expressed in rhizobia-inoculated root hairs and nodules

**Figure 2:**
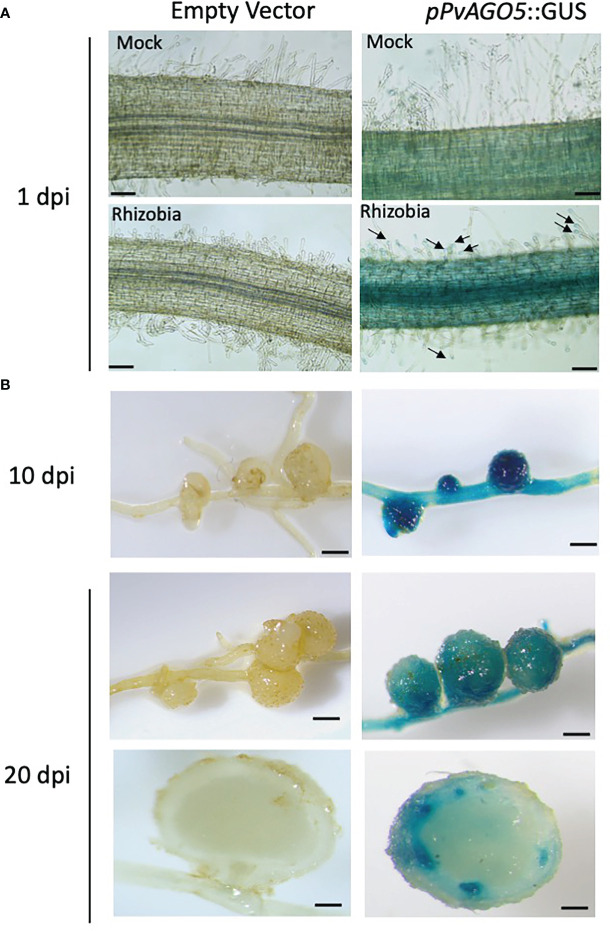
*PvAGO5* is expressed in nodule primordia and mature nodules. *P. vulgaris composite* plants with transgenic roots expressing either the empty vector or the construct *pPvAGO5:GUS-GFP* were grown under low-nitrogen conditions and inoculated with *R. tropici* CIAT899. One **(A)**, ten and twenty days after inoculation with rhizobia, transgenic roots showing rhizobia-induced deformed root hairs **(A)**, or containing nodule primordia (**B**, middle panel) or mature nodules (**B**, last four panels) were collected and stained for three hours at 37°C. Roots expressing the *pPvAGO5:GUS-GFP* construct displayed GUS signal throughout the whole root and in the tip of root hairs (**A**, black arrows). A strong GUS signal was detected in nodule primordia and in mature nodules (**B**, middle panel). Scale bars represent 100 μm for pictures shown at panel A, and 1 mm for figures shown at panel B. Images shown are representative of ten biological replicates, each one containing ten transgenic roots.

We previously demonstrated by RT-qPCR that the expression of *PvAGO5* increases at one and three hours after inoculation with rhizobia as compared to non-inoculated roots, as well as in mature nodules (Reyero-Saavedra et al., 2017). To further understand the cellular expression pattern of *PvAGO5* across different stages of root nodule symbiosis, we evaluated its spatio-temporal promoter activity in common bean composite plants. These plants were inoculated with *R. tropici* and transgenic roots were harvested one, ten, and twenty days after inoculation to evaluate the *pPvAGO5:GUS* activity during the infection process, nodules primordia at the nodule emerging stage (nodule primordia), and in mature nodules, respectively. We observed no GUS activity in mock- and rhizobia-inoculated transgenic roots, or nodules expressing the empty vector (**Figures 2A, B**). Mock-inoculated *pPvAGO5:GUS-GFP* transgenic roots showed a weak GUS signal in the whole root (**Figure 2A**). After one day of rhizobial inoculation, *PvAGO5* is expressed in the entire root (**Figure 2A**). At this time-point, we also observed a faint but consistent GUS signal in the tips of root hairs from 60 out of 80 transgenic roots expressing the *pPvAGO5:GUS-GFP* construct (**Figure 2A**), whereas the other 20 roots showed no GUS activity in these cells (**Supplementary Figure 2**). Furthermore, at ten- and twenty-days post-inoculation with rhizobia, we observed a strong GUS activity in nodule primordia, mature nodules, and throughout the vasculature system (**Figure 2B** and **Supplementary Figure 2**). Altogether, our results show that *PvAGO5* expression is increased at early stages of the root nodule symbiosis. In addition, they demonstrate that the transcriptional activity of *PvAGO5* is further enhanced during the nodule development process.

### Transcriptomic changes in *PvAGO5*-RNAi roots and mature nodules

To analyze global transcriptomic effects of *PvAGO5* down-regulation, empty vector (control) and *PvAGO5*-RNAi were analyzed from mock or *R. tropici*-inoculated roots at one day after inoculation and twenty-days-old nodules. For each time point, three biological replicates were analyzed using RNA-seq.

Overall, 10.7 to 12.2 million short sequence reads (2 X 75 bp) were generated from each of 18 RNA-seq libraries, with alignment rates to the reference genome ranging between 84-88%. Principal component analysis (PCA) showed a clustering of biological replicates and silencing-dependent variation, with the first component explaining an average of ~54% of data variation (**Supplementary Figure 3**). Comparison of expression levels in each biological condition and at each time point (relative to the empty vector) revealed 2,295 differentially expressed genes (DEGs) with significant change in expression (adjusted *P <* 0.05), including 1,497 and 798 genes that were up- or down-regulated relative to empty vector, respectively (**Supplementary Tables S3**-**S7**). This transcriptional analysis confirmed the gene silencing of *PvAGO5* (two-fold reduction) in *PvAGO5*-RNAi transgenic roots and nodules. Furthermore, we observed that the expression of other AGO protein-encoding genes was not affected by the *PvAGO5*-RNAi construct (**Supplementary Table 2**), which indicates that the gene silencing of *PvAGO5* was specific. To validate these transcriptional data sets, we randomly selected 11 genes and their expression levels were assessed by RT-qPCR. The results show similar trends between RNA-seq and RT-qPCR data (**Supplementary Figure 4**).

### *PvAGO5*-RNAi roots show increased expression of cell wall- and jasmonic acid biogenesis-related genes, but decreased expression of transporter-encoding genes under non-symbiotic conditions

**Figure 3:**
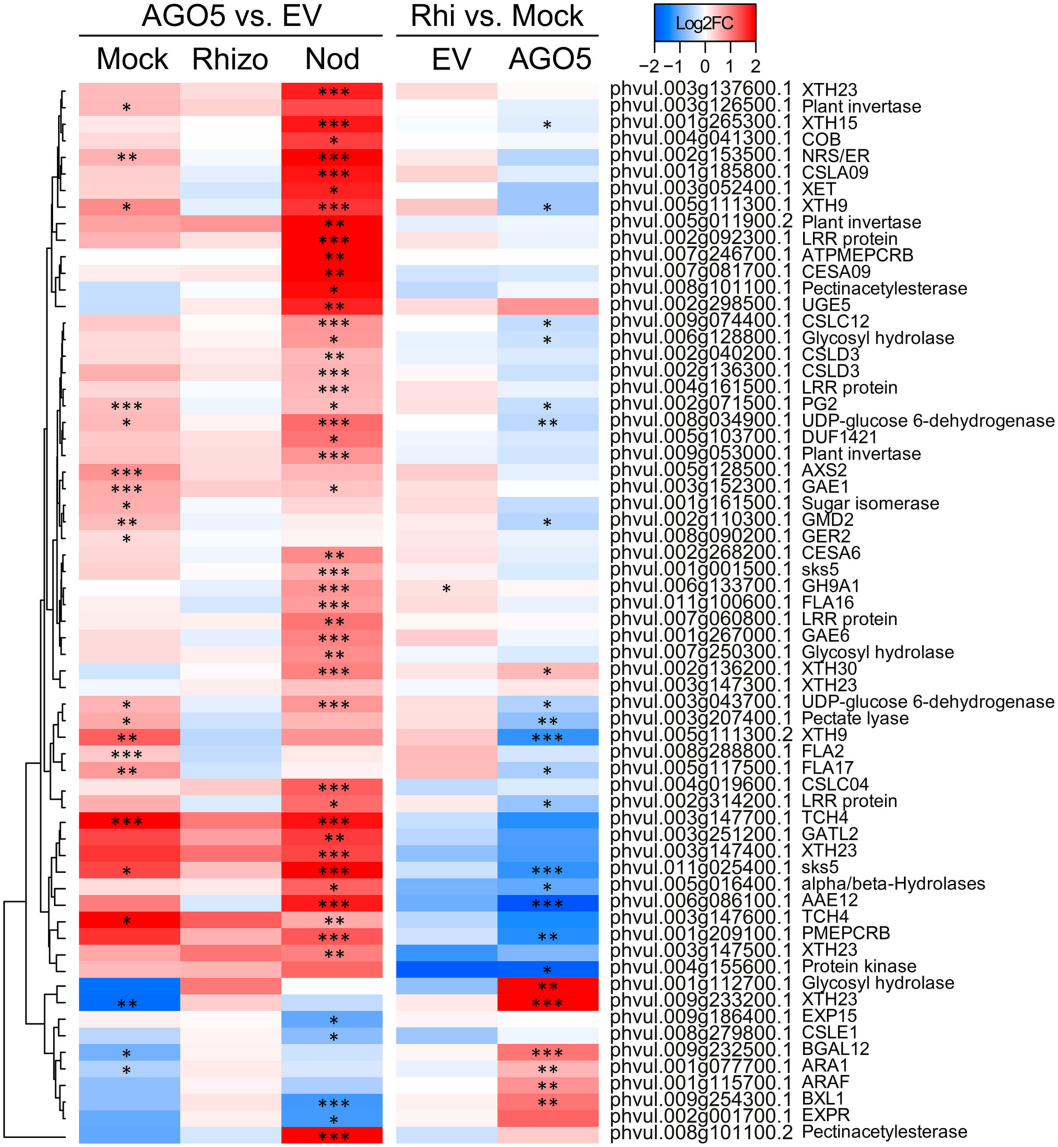
PvAGO5 modulates the expression of genes involved in the cell wall biosynthesis. Heatmap showing Log2 fold-change of gene transcripts involved in the biosynthesis and remodeling of the cell wall. Genes showing higher and lower expression difference are shown in different shades of red and blue, respectively. Labels at the top of the heatmap located on the left side indicate: Mock= uninoculated transgenic roots; Rhizo= rhizobia-inoculated transgenic roots, and Nod = mature nodules. In both cases the comparison between *PvAGO5-*RNAi vs Empty Vector is shown. Labels at the top of the heatmap located on the right side indicate: EV= transgenic roots expressing the empty vector (control), and AGO5: transgenic roots expressing the *PvAGO5*-RNAi construct. In both cases the comparison of rhizobia-inoculated roots vs mock-inoculated roots is shown. Asterisks indicate different levels of statistical significance of the comparisons (*: adjusted P-value<0.05; **= adjusted P-value<0.01; ***= adjusted P-value<0.001). Genes with no asterisk are not significantly differentially expressed. Dendrogram (left) represents the transcript clustering based on expression profile.

To gain insight into the role of AGO5 under non-symbiotic conditions, we compared the transcriptomes of transgenic roots carrying the *PvAGO5*-RNAi against the empty vector in the mock samples. This analysis led to the identification of 962DEGs, 598 up-regulated and 364 down-regulated genesin*PvAGO5*-RNAi roots, respectively (**Figure 3** and **Supplementary Table 3**). Gene Ontology term enrichment analysis of the identified DEGs revealed that the most significant GO terms were those involved in cell wall biogenesis (**Supplementary Table 8**). Their upregulation in *PvAGO5*-RNAi transgenic roots suggests that AGO5 may play a role in negatively regulating the biogenesis of the cell wall during root development.

**Figure 4:**
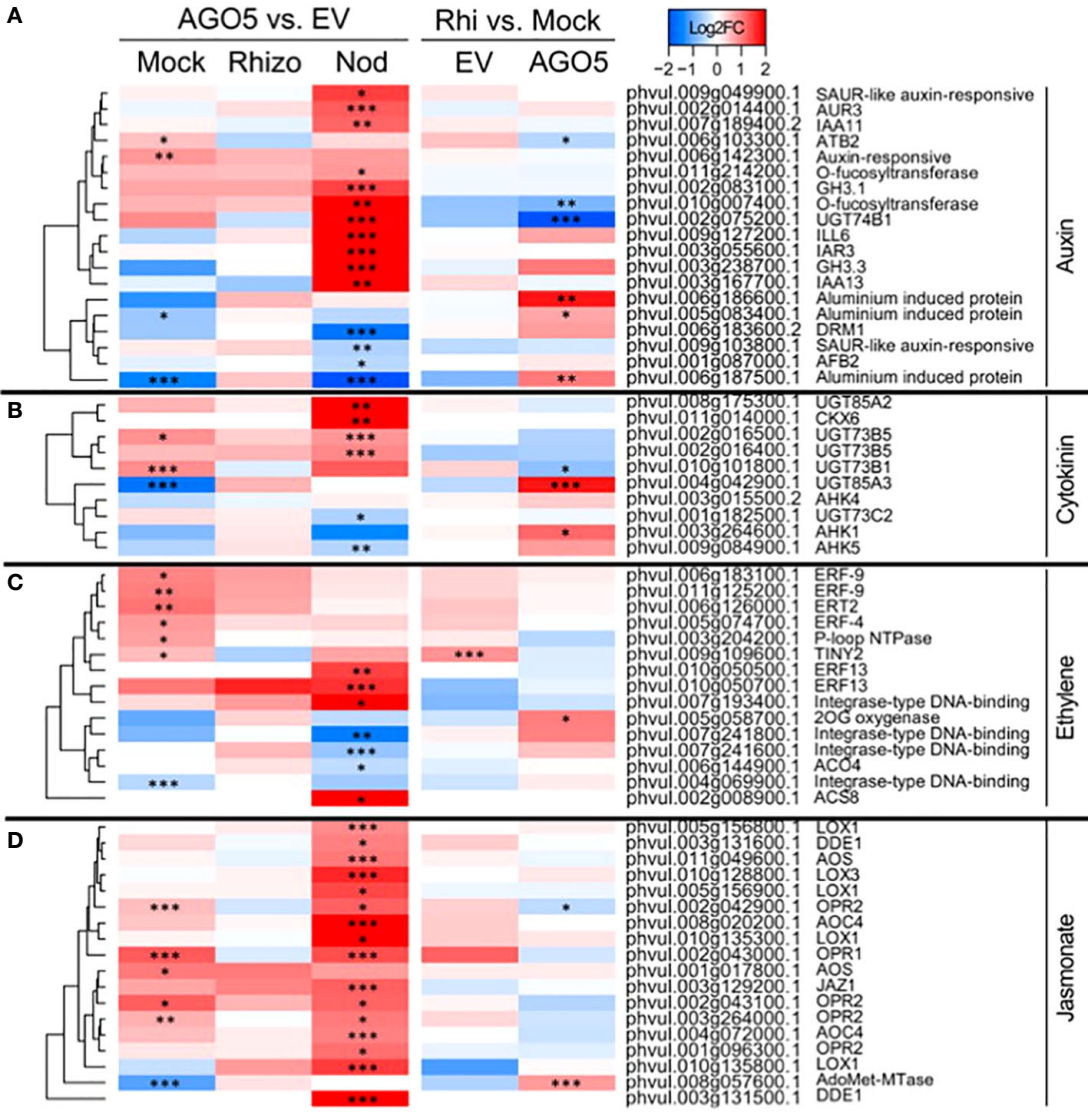
PvAGO5 modulates the expression of genes participating in the biosynthesis and signaling of phytohormones. Heatmaps showing Log2 fold-change of gene transcripts involved in the biosynthesis and signaling of auxins **(A)**, cytokinins **(B)**, ethylene **(C)**, and jasmonate **(D)**. Fold-changes are false-colored. Genes showing higher and lower expression difference are shown in different shades of red and blue, respectively. Labels at the top of the heatmap located on the left side indicate: Mock= uninoculated transgenic roots; Rhizo= rhizobia-inoculated transgenic roots, and Nod = mature nodules. In both cases the comparison between *PvAGO5-*RNAi vs Empty Vector is shown. Labels at the top of the heatmap located on the right side indicate: EV= transgenic roots expressing the empty vector (control), and AGO5: transgenic roots expressing the *PvAGO5*-RNAi construct. In both cases the comparison of rhizobia-inoculated roots vs mock-inoculated roots is shown. Asterisks indicate different levels of statistical significance of the comparisons (*: adjusted P-value<0.05; **= adjusted P-value<0.01; ***= adjusted P-value<0.001). Genes with no asterisk are not significantly differentially expressed. Dendrogram (left) represents the transcript clustering based on expression profile.

Further KEGG pathway functional classification not only confirmed the participation of AGO5 in cell wall biogenesis, but also revealed that gene silencing of *PvAGO5* increases the expression of genes related to the jasmonic acid biosynthesis and ethylene perception (**Figures 4C, D** and **Supplementary Table 9**). This functional classification analysis also indicated that the downregulation of *PvAGO5* diminished the expression of genes encoding diverse types of transporters, among them auxin-, cation-, carbohydrate- and amino acid transporters (**Supplementary Figure 5**). Altogether, this transcriptional analysis implicates PvAGO5 in root development, likely through the regulation of genes involved in cell wall biogenesis and in the modulation of multiple phytohormones.

### *PvAGO5*-RNAi roots show increased expression of defense-related genes during the first day of rhizobial interaction

**Figure 5:**
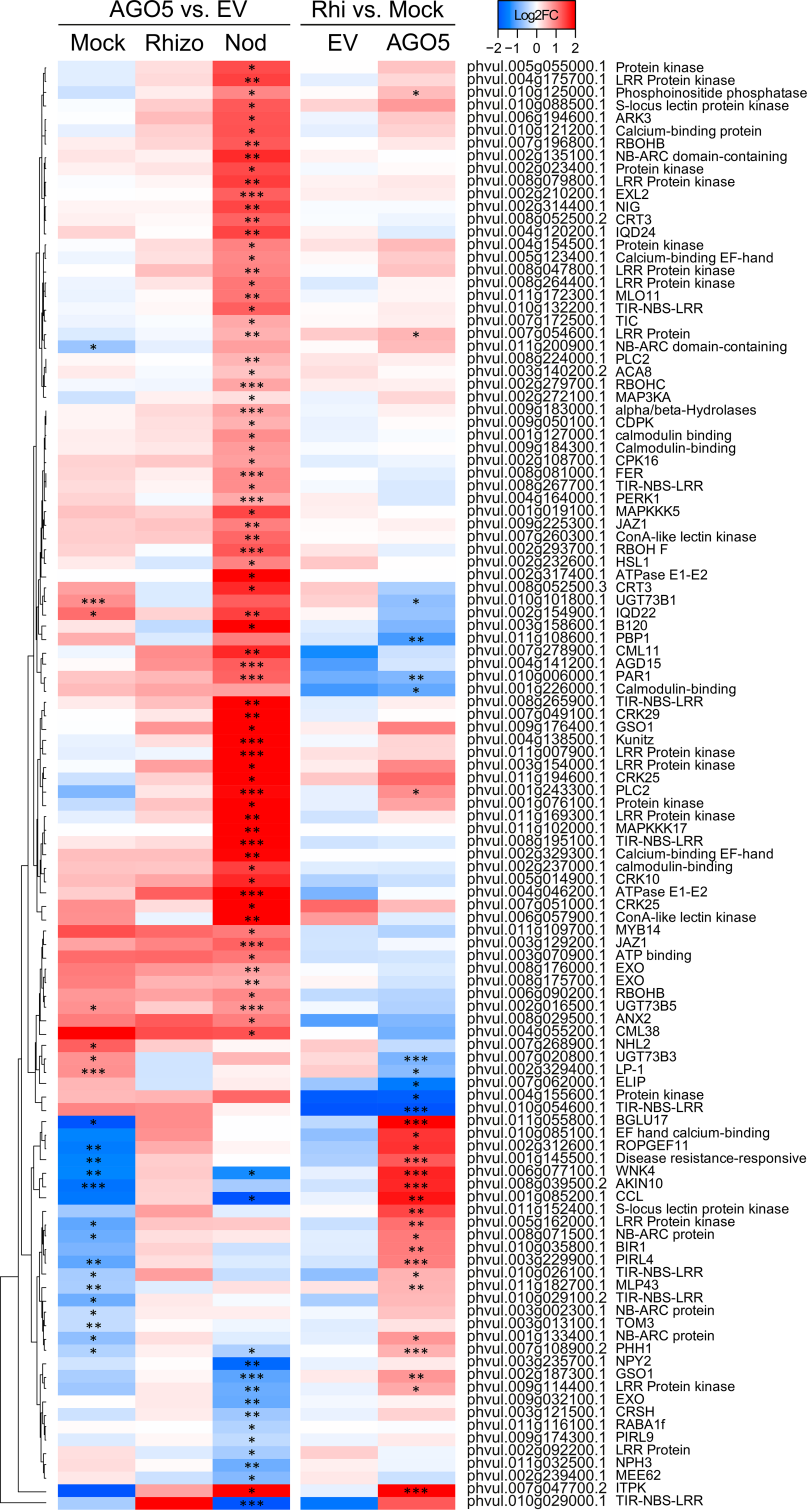
Down-regulation of *PvAGO5* increases the expression of plant-defense related genes. Heatmap showing Log2 fold-change of gene transcripts involved in plant defense response in uninoculated- (Mock) and rhizobia-inoculated roots (rhizobia), as well as in mature nodules (Nod). Genes showing higher and lower expression differences are shown in different shades of red and blue, respectively. Labels at the top of the heatmap located on the left side indicate: Mock= uninoculated transgenic roots; Rhizo= rhizobia-inoculated transgenic roots, and Nod = mature nodules. In both cases the comparison between *PvAGO5-*RNAi vs Empty Vector is shown. Labels at the top of the heatmap located on the right side indicate: EV= transgenic roots expressing the empty vector (control), and AGO5: transgenic roots expressing the *PvAGO5*-RNAi construct. In both cases the comparison of rhizobia-inoculated roots vs mock-inoculated roots is shown. Asterisks indicate different levels of statistical significance of the comparisons (*: adjusted P-value<0.05; **= adjusted P-value<0.01; ***= adjusted P-value<0.001). Genes with no asterisk are not significantly differentially expressed. Dendrogram (left) represents the transcript clustering based on expression profile.

Our spatio-temporal analyses indicated that *PvAGO5* is expressed in root hairs in response to rhizobia, suggesting that this AGO plays a role in the rhizobial infection process. To evaluate this hypothesis, we analyzed the transcriptional responses of *PvAGO5*-RNAi transgenic roots during the first day of interaction with *R. tropici*. Our transcriptome analyses revealed no overall drastic changes in the expression of canonical genes involved in the molecular dialogue between both symbionts and the rhizobial infection process when compared with rhizobia-inoculated transgenic roots expressing the empty vector. Instead, this transcriptome analysis revealed that the expression of genes related to plant defense, which must be modulated to allow the rhizobial infection (Berrabah et al., 2015; Wang et al., 2016; Berrabah et al., 2019), was significantly increased in *PvAGO5*-RNAi transgenic roots during the first day of interaction with rhizobia (**Figure 5**). We observed that the expression of genes encoding NBC-ARC domain-containing disease resistance proteins (e.g., Phvul.008g071500.1 and Phvul.001g133400.1) or LRR Receptor-Like Serine/Threonine-Protein Kinase (e.g., Phvul.005g162000.1 and Phvul.002g187300.1) increased two-fold in response to rhizobia (**Figure 5**). These transcriptional data indicate that the downregulation of *PvAGO5* activates the expression of plant defense-related genes in response to rhizobia.

### The expression of phytohormone-, plant defense-, and cell wall biogenesis-related genes is affected in mature *PvAGO5*-RNAi nodules

Gene silencing of AGO5 significantly reduces nodule size and the number of rhizobial-infected nodule cells (Reyero-Saavedra et al., 2017). To explore the basis of this phenotype, we analyzed the transcriptome of mature nodules expressing the *PvAGO5*-RNAi or the empty vector. This analysis led us to the identification of 1,006 DEGs, of which 766 were upregulated and 240 downregulated in *PvAGO5*-RNAi nodules.

Functional classification of the DEGs indicated that many of the up-regulated genes were related to plant defense (**Figure 5**). For example, we found that oxidative burst-associated genes (i.e., *PvRbohA*: Phvul006g090200.1) or genes encoding resistance and pathogenesis-related (PR) proteins were significantly upregulated in *PvAGO5*-RNAi nodules (**Figure 5**). Interestingly, we also identified a three-fold induction of an *Rj4* orthologue (Phvul.002g107900) which encodes a thaumatin-like pathogenesis-protein known to restrict nodulation to specific rhizobial strains in soybean (**Supplementary Figure 6** and **Supplementary Table 7**) (Tang et al., 2016).

Silencing of *PvAGO5* in mature nodules resulted in the significant upregulation of genes involved in the biosynthesis and signaling of the phytohormones auxin, ethylene, and jasmonic acid (**Figure 4** and **Supplementary Table 9**). In contrast, genes involved in cytokinin degradation were up-regulated, whereas genes participating in its perception and signaling were downregulated in mature *PvAGO5*-RNAi nodules (**Figure 4** and **Supplementary Table 9**).

Furthermore, we observed that the expression of genes participating in cell wall biogenesis and remodeling significantly increased (**Figure 3** and **Supplementary Table 7**, **8**). However, we also observed that some genes belonging to this category were downregulated. For instance, the expression of the gene *Increasing Nodule Size1* (*INS1*: Phvul.003g016300.1), which encodes for a UDP-Glycosyltransferase/trehalose-phosphatase, was significantly diminished in *PvAGO5*-RNAi nodules (**Supplementary Figure 6** and **Supplementary Table 7**). Altogether, these transcriptional data indicate that PvAGO5 regulates nodule development and functioning by modulating the expression of genes involved in the phytohormone balance, control of the plant defense response, and cell wall biogenesis, which is determinant for this symbiosis.

### The expression of mineral nutrient transporter-related genes is affected in *PvAGO5*-RNAi mature nodules

Legume hosts constantly translocate different mineral nutrients to the nodules to sustain an effective symbiosis. Defects in this mineral nutrient exchange compromise the symbiosis (Liu et al., 2020; Castro-Rodríguez et al., 2021). Hence, we investigated whether *PvAGO5* downregulation affects the expression of genes encoding mineral nutrient transporters in mature nodules. We found that the expression of genes encoding Fe^2+^-, Mn^2+^, and K^+^ transporters was significantly diminished in *PvAGO5*-RNAi mature nodules (**Supplementary Figure 5**). We also observed that the expression of Phvul.007g275300.1 that encodes the PHO1 transporter, which is required to transfer phosphate from infected nodule cells to bacteroids (Nguyen et al., 2021), was significantly downregulated. In contrast, genes encoding different ABC and MATE transporter family members, as well as sugars- and amino acid- transporters, were upregulated. Altogether, these transcriptional data indicate that PvAGO5 regulates nodule functioning by modulating the expression of genes involved in mineral nutrient transport underpinning the symbiosis.

### The sRNA cargo of PvAGO5 is dynamic during root nodule development

To obtain further insights into the roles of PvAGO5 in the root nodule symbiosis and to determine changes in sRNA associations during this symbiosis, we isolated, sequenced, and compared PvAGO5-bound sRNA pools from nodule primordia and mature nodules. We choose mature nodules because of the strong transcriptional activity of the *PvAGO5* promoter during this stage of development. We also included nodule primordia and non-inoculated roots to capture those sRNAs that accumulate during nodule development. For each experimental condition, we included three biological replicates. For downstream analyses, we focused only on those sRNAs present in the three libraries from each of the three experimental conditions.

**Figure 6:**
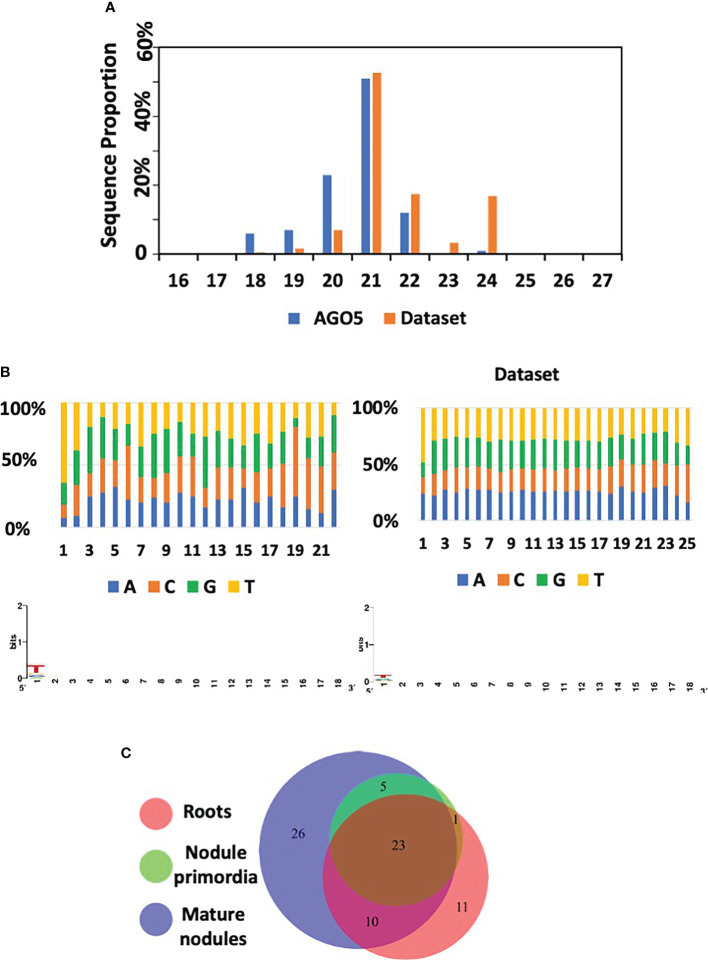
AGO5 sRNA cargo dynamically changes through the nodule development. **(A)** Length distribution of mapped sRNA reads isolated from AGO5 and the corresponding starting dataset. Starting dataset refers to the set of small RNA sequences used as reference for annotation of the sequencing results. **(B)** Read nucleotide distribution and 5’ bias of AGO5-associated sRNAs. **(C)** Number of sRNA associated in AGO5 from uninoculated roots, roots bearing nodule primordia, and mature nodules.

PvAGO5-associated sRNAs consisted of 18-22 nt sRNAs, with a 5’ C and U bias, and an enrichment and a depletion of the sequence shorter and longer than 21-nt, respectively, compared to the starting dataset (**Figures 6A, B**). We identified 76 sRNAs, 72 of them were miRNAs and the rest rhizobial-derived tRFs. Sixty-nine of the identified miRNAs were grouped into 26 known miRNA families. The other three miRNAs were classified as *P. vulgaris*-specific miRNAs. 50% of these 72 miRNAs were grouped into only seven families, corresponding to miR156, miR159, miR166, miR167, miR168, miR319, and miR396. (**Supplementary Table 10**).

A comparison between the PvAGO5-bound sRNAs from the three experimental conditions revealed that 23 out of the 76 sRNAs were present in the three tested biological samples (uninoculated roots, root bearing nodule primordia, and mature nodules), whereas the rest were present in at least two biological conditions (i.e., present in roots and in nodule primordia) or unique for a particular condition (i.e., specific to mature nodules) (**Figure 6C**). Among the 23 miRNAs present in the three experimental conditions, Pvu-miR482a-5p, Pvu-miR166a-3p, Gmax-miR1511, gma-miR6300, Pvu-miR166h-3p, Pvu-miR396c-5p, Pvi-miR156a-5p, Gmax-miR156a, Pvu-miR396b-5p, Pvu-miR1511-3p, Cme-miR168, hbr-miR156, and Pvu-miRN95, were the most abundant miRNAs (**Figure 6** and **Supplementary Table 10**). Most of these microRNA families are known to be involved in the regulation of pathways that are found altered in our transcriptomic data analysis between *PvAGO5*-RNAi and EV plants and could therefore explain the observed phenotypic. Some of these families are directly related to nodulation such as miR482, miR166 and miR156 (Boualem et al., 2008; Li et al., 2010; Wang et al., 2015). In the case of miR156 and miR396 families, they are involved in flavonoid biosynthesis (Yang et al., 2022) which could partly explain that, when we alter the production of AGO5, these microRNAs being associated, the biosynthesis of flavonoids is altered. Finally, an important candidate for the explanation of nodulation alteration is miR1511, that has been shown to regulate root growth under abiotic stresses, *via* iron homeostasis and ROS accumulation in roots (Martin-Rodriguez et al., 2021). As iron and ROS are components regulating rhizobial symbiosis at different stages of its establishment, it is likely that, by altering AGO5, we alter the function of miR1511, the corresponding iron and ROS accumulations and, ultimately, nodulation.

We found eleven, zero, and twenty-six miRNAs that were uniquely bound to PvAGO5 from uninoculated roots, roots bearing nodule primordia, and mature nodules, respectively (**Supplementary Table 11**). Among the 26 unique sRNAs bound to PvAGO5 from mature nodules, four of them were tRFs from *R. tropici*, which represents a 33-times enrichment compared to the original dataset (**Supplementary Table 11**). Interestingly, this type of sRNAs was recently shown to play a role in the root nodule symbiosis in soybean (Ren et al., 2019).

Next, we predicted putative target genes of AGO5-bound sRNAs by using psRNAtarget V2 using default parameters (Dai et al., 2018). Sixty-four out of 76 identified sRNA were predicted to target at least one mRNA (**Supplementary Table 12**). Although no GO terms nor any pathway were significantly enriched in the identified target gene set, analysis of protein domain enrichment reveals that AGO5-bound sRNAs preferentially target genes containing domains related to defense (e.g., NB-ARC, Leucine-rich repeat and Toll/interleukin-1 receptor), growth and development process (e.g., ZPR1-type TFs, SPB-box TFs, and Growth-regulating factor) or nodulation (e.g., Nodulin) (**Supplementary Table 13**). Next, we analyzed the expression of the predicted target genes in our transcriptome data set. Interestingly, the majority of the predicted target genes were up-regulated instead of the canonical down-regulation expected for sRNA target genes (**Supplementary Table 12**).

In summary, these data indicate that PvAGO5 can bind sRNA, both plant- and rhizobia-derived, that may play a role in the nodule development and functioning. The fact that the predicted target genes were up-regulated suggests that AGO5 may play a role in negatively regulating the protein production of sRNA targets, as well as the principal miRNA effector protein AGO1. However, further investigation is required to confirm this hypothesis.

## Discussion

**Figure 7:**
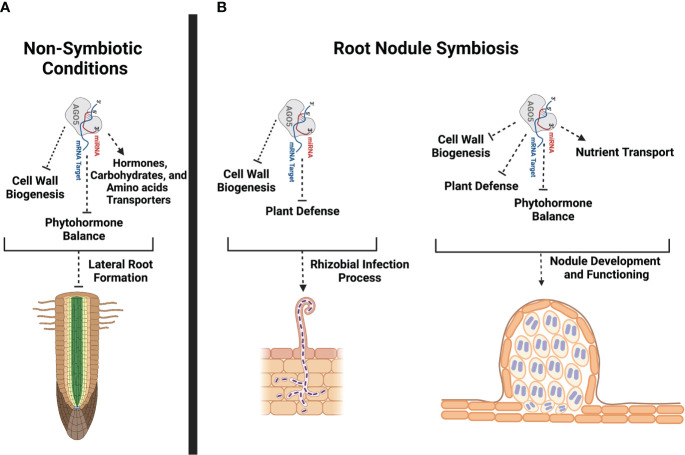
Schematic representation of potential PvAGO5 roles in lateral root formation and root nodule symbiosis. **(A)** Based on transcriptional data and phenotype from *PvAGO5*-RNAi transgenic roots growing under non-symbiotic conditions, PvAGO5 might modulates the expression of genes involved in the cell wall biogenesis, phytohormone balance and transportation. Modulation of the expression of these genes may be required to control the number and density of lateral roots in *P. vulgaris*. **(B)** In contrast, AGO5 acts as a positive regulator of the root nodule symbiosis in *P. vulgaris*. PvAGO5 modulates the expression of genes participating in the flavonoid biosynthesis, cell wall remodeling, and plant defense. It is likely that PvAGO5 participates in the cell wall remodeling, which is required to sustain the root hair curling that is crucial for rhizobial entrapping. Likewise, PvAGO5 may modulate the plant defense response to ensure the rhizobial infection and colonization. At late stages of this symbiosis, PvAGO5 may participate in cell wall remodeling and phytohormone balance allowing a proper nodule formation. Additional, PvAGO5 may also contribute to the nodule functioning by controlling the expression of nutrient transporter-encoding genes. Finally, it is likely that AGO5 also modulates the plant defense to host rhizobia during the nitrogen-fixation process. This figure was created with Biorender.com.

sRNAs recruited into the multiprotein complex RISC play an important role in regulating the root nodule symbiosis in diverse legumes. However, to date, the role of AGOs, the main protein component of RISC, in this symbiosis has been overlooked. Publicly available transcriptional data indicate that *PvAGO1*, *PvAGO5*, and *PvAGO10*, the genes coding for the three AGOs constituting a phylogenetic clade, are expressed in different *P. vulgaris* tissues, including leaves, shoots, pods, roots, and nodules (**Supplementary Figure 8**). *PvAGO1* is the most expressed, followed by *PvAGO5* and *PvAGO10*, except in the shoots where *PvAGO10* is more expressed than *PvAGO5*. Neither correlation nor contrast in the expression of these genes seems to occur in the analyzed tissues. Proportionally to the expression of *PvAGO1*, *PvAGO5* shows its greatest relative expression in effective nodules, compared to other tissues, and its accumulation is systematically greater in nodules or inoculated roots compared to the corresponding controls (**Supplementary Figure 8**). We previously confirmed by RT-qPCR that the expression of *PvAGO5* increases in nodules compared to leaves and uninoculated roots (Reyero-Saavedra et al., 2017). This transcriptional data suggested that PvAGO5 plays a role in the root nodule symbiosis. Indeed, we previously showed that the silencing of *AGO5* negatively affects nodule formation and reduces the number of rhizobia-infected nodule cells in *G. max* and *P. vulgaris* (Reyero-Saavedra et al., 2017). In this study, we provide additional transcriptional evidence supporting the notion that AGO5 modulates the expression of diverse genes involved in the rhizobial infection process, nodule development, and nodule functioning (**Figure 7**).

AGO5 and, therefore, its cargo sRNAs play different physiological and developmental roles. For instance, AGO5 modulates part of the plant defense responses against the *Potato virus X* and aphids in *A. thaliana* and wheat, respectively (Brosseau and Moffett, 2015; Sibisi and Venter, 2020). AGO5 also participates in the seed coat pigmentation in *G. max* (Cho et al., 2017). Additionally, AGO5 controls flowering time by modulating the expression of the transcription factor *Squamosa Binding Protein-Lik*e (*SPL*) through the action of miR156 in *A. thaliana* (Roussin-Léveillée et al., 2020). Recent evidence also indicates that AGO5 along with AGO9 bind to transposon-derived sRNAs to mark an early-segregating germline in *A. thaliana* (Bradamante et al., 2022). In this study, we provide a range of evidence for the participation of AGO5 in several aspects of root nodule symbiosis in *P. vulgaris*. This conclusion is supported by the expression of *PvAGO5* during the rhizobial infection and throughout the nodule development process. Furthermore, the downregulation of *PvAGO5* activates the expression of genes related to the plant defense response and the biosynthesis of phytohormones that negatively affects this symbiosis, as previously reported in both *P. vulgaris* and *G. max* (Reyero-Saavedra et al., 2017) (**Figure 7**).

There is mounting evidence that *AGO5* is expressed in egg and sperm cells of mature gametophytes, developing carpels, axillary meristems, the subepidermal layer of the shoot apical meristem, and in the root apical meristem of *A. thaliana* (Borges et al., 2011; Tucker et al., 2012; Bradamente et al., 2022; Jullien et al., 2022). Based on the *A. thaliana* transcriptome atlas (https://www.arabidopsis.org), *AGO5* is also expressed in the xylem pole pericycle during lateral root initiation. In this study, we observed that *PvAGO5* is expressed in the lateral root primordium and throughout lateral root under non-symbiotic conditions. Additionally, the downregulation of *PvAGO5* led to an 15% increase in the number and density of lateral roots (**Figures 1I–K**). Hence, our data support earlier observations indicating that *AGO5* is active in root meristems, and we provide new evidence suggesting that AGO5 plays a role in lateral root formation.

Our transcriptome analysis of uninoculated *PvAGO5*-RNAi transgenic roots revealed that the expression of genes participating in jasmonic acid and cell wall biogenesis is increased when compared with control roots. Jasmonic acid is an essential phytohormone for the activation of the plant defense response. However, this phytohormone also promotes lateral root formation in a dose-dependent manner in *A. thaliana* (Sun et al., 2009; Raya-González et al., 2012; Cai et al., 2014). To sustain root growth, the cell wall must be modified and rebuilt during this process (Somssich et al., 2016). Hence, our transcriptional data indicate that the increase in the expression of genes associated with jasmonic acid and cell wall biogenesis may promote and sustain the formation of lateral roots observed in *PvAGO5*-RNAi transgenic roots. However, the role and mechanism of action of PvAGO5 in the formation of lateral roots need to be experimentally validated (**Figure 7**).

In the root nodule symbiosis context, we detected a specific expression of *PvAGO5* in the root hair tips at one-day post-inoculation with rhizobia (**Figure 2**). At this stage, root hair cell wall is softened allowing the entrance of rhizobia *via* the so-called infection thread (van Spronsen et al., 1994). Our transcriptomic data shows a significant increase in the expression of cell wall biosynthesis-related genes in *PvAGO5*-RNAi transgenic roots, indicating a role of PvAGO5 in their regulation. Altogether, these observations confirm that PvAGO5 plays a role in the early rhizobial infection process. Furthermore, these data provide lines of evidence that explain the reduced rhizobial infection observed in *PvAGO5*-RNAi transgenic roots (Reyero-Saavedra et al., 2017). This phenotype may be partly attributed to the lack of PvAGO5-mediated regulation of cell wall biosynthesis-related genes at the infection pocket and infection thread initiation site. This defect may prevent the proper cell wall softening and rhizobial infection (**Figure 7**).

At late stages of the root nodule symbiosis, we observed that *PvAGO5* is also expressed in nodule primordia and mature nodules. These observations add more evidence supporting the notion that AGO5 is determinant in the nodule development. This hypothesis is supported by the fact that the downregulation of *PvAGO5*-RNAi results into a significant reduction in the number and size of nodules in *P. vulgaris* (Reyero-Saavedra et al., 2017). Studies in different legumes have identified different genetic components that regulate nodule development and size. For example, *INS1*, which encodes a cell wall β-expansin and is expressed in nodules, is a regulator of nodule development in *G. max* by positively controlling the enlargement of nodules and infection cells (Li et al., 2018). In this study, we found that the expression of *INS1* was significantly diminished in *PvAGO5*-RNAi mature nodules. Hence, the reduction in the number of nodules and their size, observed in *PvAGO5*-RNAi composite plants, can partially be explained by the altered expression of this regulator.

During the root nodule symbiosis, the plant defense response is finely tuned. Mounting evidence indicates that a sustained plant defense response compromises this symbiosis (Berrabah et al., 2015; Berrabah et al., 2019). In this study, we observed that genes related to the plant defense were significantly upregulated and enriched in rhizobia-inoculated *PvAGO5*-RNAi transgenic roots and mature nodules. One of these genes were *Rj4*, which encodes a Thaumatin-like protein (Hayashi et al., 2014). Rj4 restricts the rhizobial infection process and nodule development in incompatible interactions between *G. max* and rhizobia (Hayashi et al., 2014). Similarly, our transcriptomic analyses also revealed that the expression of flavonoid biosynthesis-related genes was significantly upregulated in *PvAGO5*-RNAi transgenic roots and nodules (**Supplementary Figure 7**). Flavonoids participate in different steps of the root nodule symbiosis, from early secreted signals to auxin balance during nodule organogenesis (Liu and Murray, 2016). Flavonoids also have a role in the plant resistance against bacterial infection (Mierziak et al., 2014) Hence, it is likely that the activation of the plant defense response may compromise the establishment of the root nodule symbiosis, particularly affecting the rhizobial infection process in *P. vulgaris*. This hypothesis is supported by the observation that *AGO5* down-regulation affects the rhizobial colonization in both root and nodule cells in *P. vulgaris* and *G. max* (Reyero-Saavedra et al., 2017).

Phytohormones play a determinant role in each step of the root nodule symbiosis (Lin et al., 2020). According to studies across diverse legumes, in an optimal balance, auxin and cytokinin are positive regulators of this symbiosis (Lin et al., 2020). For instance, cytokinins are required for nodule organogenesis and development (Heckmann et al., 2011; Reid et al., 2017). Additionally, disruption of auxin transport inhibits nodule formation (Huo et al., 2006). In contrast, several lines of evidence indicate that ethylene, jasmonic acid, and abscisic acid negatively affect the establishment of the root nodule symbiosis (Lin et al., 2020). Here, we observed that the down-regulation of *PvAGO5* decreases the expression of genes involved in auxin and cytokinin biosynthesis and signaling in rhizobia-inoculated roots. In contrast, the expression of genes participating in the biosynthesis and signaling of jasmonic acid and ethylene was significantly increased in *PvAGO5*-RNAi rhizobia-inoculated roots and nodules. Furthermore, it has been demonstrated that the miR319d/TCP10 node regulates the rhizobial infection process through the modulation of genes involved in the jasmonic acid biosynthesis in *P. vulgaris* (Martín-Rodríguez et al., 2018). Reduction in the miR319d level led to a significant reduction in the number of rhizobial-induced deformed root hairs and nodules (Martín-Rodríguez et al., 2018). Our data reveals that miR319 is one of the families most associated with AGO5, linking the jasmonic acid-related transcriptomic response, and the observed phenotype, to AGO5 cargo. Thus, the reduction in both rhizobial infection and number of nodules previously observed (Reyero-Saavedra et al., 2017) can be explained by the activation of jasmonic acid biosynthesis- and signaling related genes, likely due to the lack of miR319/AGO5 association.

Effective root nodule symbiosis depends on efficient nutrient exchange. Recent studies indicate that the Phosphate Transporter1.1 (PHO1.1) and PHO1.2 translocate phosphate from the infected cell to nitrogen-fixing bacteroids in *M. truncatula* (Nguyen et al., 2021). Similarly, different types of iron transporters, among them Yellow Stripe-Like and Vacuolar Iron Transporter Like, aid the translocation of iron from infected nodule cells to nitrogen-fixing bacteria (Liu et al., 2020; Castro-Rodríguez et al., 2021). Nutrient transporter failure negatively affects the functioning of mature nodules (Liu et al., 2020; Castro-Rodríguez et al., 2021; Nguyen et al., 2021). In this study, we observed that the expression of genes coding for mineral nutrient transporters, including different iron transporters and the phosphate transporter PHO1, was diminished in both *PvAGO5*-RNAi rhizobia-inoculated roots and nodules. The downregulation of these mineral nutrient transporters, essential for nodule functioning, by the repression of *PvAGO5* suggests that is likely that PvAGO5 sustains an effective root nodule symbiosis by modulating the expression of mineral nutrient transporters in mature nodules.

Studies in different legumes attest to the relevance of miRNAs in nodule development (Tiwari et al., 2021). All the phenotypic and transcriptomic alterations observed in *PvAGO5*-RNAi transgenic roots, in a nodulation context or not, are likely derived from the sRNA-guided AGO5 activity. Here, we found that 19 of the 26 conserved miRNAs cargo of PvAGO5 (e.g.: miR156, miR166, miR169, miR171, miR172, miR319, miR390, miR393, and miR482) have previously been demonstrated to play a role in regulating the rhizobial symbiosis and/or modulating the plant defense response (Tiwari et al., 2021). However, most of the target genes predicted for the identified sRNAs, which display a significant differential expression in *PvAGO5*-RNAi roots compared to EV, are not canonical targets and are not known to participate in the symbiotic process. From the gene set of targets showing significant differential expression, we observed that around 93% of them showed an up-regulation in *PvAGO5*-RNAi mature nodules. Hence, our data suggest that sRNAs bound to AGO5 guide the negative regulation of genes required for effective root nodule symbiosis, a function similar to that of the other principal member of the same AGO clade, AGO1.

Recent evidence in *G. max* also attests the participation of rhizobia-derived tRFs in the root nodule symbiosis. tRFs were 33-fold enriched in the sRNA cargo of PvAGO5 from mature nodules. Three of them are predicted to target previously undescribed targets that are significantly up-regulated in our *PvAGO5*-RNAi root samples: *Early-Responsive to Dehydratation* (*ERD*), *Early NODulin-Like protein 17* (*ENOD17*), and *HEMA1*, a Glutamyl-tRNA reductase. The last two are likely to be related to nodulation. *ENOD17* encodes a nodulin-like protein, and *HEMA* is a homolog of *HEMA* genes encoding a Glutamyl-tRNA reductase known to participate in the heme synthesis of leghemoglobins in *L. japonicus* nodules (Wang et al., 2019). Hence, our data suggest that AGO5 and its sRNA cargo regulate the expression of diverse genes that are crucial for the rhizobial infection process as well as for nodule development and functioning. Furthermore, the role of the tRFs bound to AGO5 in the root nodule symbiosis must be experimentally tested.

## Conclusions

Overall, the data presented in this study lead us to conclude that PvAGO5 and its associated sRNA modulate the expression of genes with diverse functions that are crucial for the development and maintenance of an effective root nodule symbiosis. The observation that the downregulation of *PvAGO5* has opposite effects in the lateral root versus nodule development, suggests that this AGO modulates the expression of genes required for the developmental programs of these distinct organs according to the nitrogen needs of *P. vulgaris* (**Figure 7**).

## Data availability statement

The original contributions presented in the study are publicly available. The data presented in the study are deposited in the BioProject repository, accession number PRJNA835127 (https://www.ncbi.nlm.nih.gov/bioproject/PRJNA835127).

## Author contributions

OV-L, DF, and JR proposed and designed the study. MS-C, MI-A, EP-R, and MR-S generated common bean composite plants. EP-R and MR-S generated the *pPvAGO5::GUS* and *PvAGO5*-RNAi constructs. OV-L, MI-A and MS-C generated the transgenic tissues used in this study. OV-L, MI-A, and MS-C purified total RNA from *P. vulgaris* tissues. OV-L, MI-A, and MDSS-C performed the AGO5 immunoprecipitation and sRNA purification. AS-F and VJ-J, prepared and sequenced the mRNA and sRNA libraries. DF, AS-F, VJ-J, SC, and AM-S analyzed the RNAseq data. OV-L, MI-A, and EP-R confirmed the RNAseq data by RT-qPCR. OV-L, DF, SC, and AM-S generated figures and performed statistical analyses. OV-L, DF, and JR wrote the manuscript. All authors read and approved the manuscript.

## Funding

This work was supported by the Consejo Nacional de Ciencia y Tecnología (CONACyT grant No. A1-S-9454 and No. A1-S-16129) and by the Programa de Apoyo a Proyectos de Investigación e Inovación Tecnológica (PAPIIT-UNAM grant No. IN201320 and No. IA201522) to OV-L and DF, respectively. Research in JR laboratory is supported by a grant from PAPIIT-UNAM IN202918. MI-A is a doctoral student from Programa de Doctorado en Ciencias Biológicas, Universidad Nacional Autónoma de México, and received a doctoral student fellowship from CONACyT (CVU: 919676). SC is a doctoral student from the Programa de Doctorado en Ciencias Biomédicas, Universidad Nacional Autónoma de México, and has received CONACyT fellowship #1002252.

## Acknowledgments

We thank Dr. Caspar C.C. Chater (Royal Botanic Gardens, Kew) and Dr. Georgina Hernández (Centro de Ciencias Genomicas-UNAM, Mexico) for constructive discussion.

## Conflict of interest

The authors declare that the research was conducted in the absence of any commercial or financial relationships that could be construed as a potential conflict of interest.

## Publisher’s note

All claims expressed in this article are solely those of the authors and do not necessarily represent those of their affiliated organizations, or those of the publisher, the editors and the reviewers. Any product that may be evaluated in this article, or claim that may be made by its manufacturer, is not guaranteed or endorsed by the publisher.
