# Supplementary material for: Argonaute5 and its associated small RNAs modulate the transcriptional response during the rhizobia-Phaseolus vulgaris symbiosis
Source: Front Plant Sci. 2022 Nov 17;13:1034419. doi: 10.3389/fpls.2022.1034419 (PMC9714512; doi:10.3389/fpls.2022.1034419)
Supplement: Supplementary file 1 [file DataSheet_1.zip › Supplementary Figures.pdf]

Supplementary Material for:

Argonaute5 and its associated small RNAs modulate the rhizobial infection and nodule development in *Phaseolus vulgaris*

María del Socorro Sánchez-Correa[1], Mariel C. Isidra-Arellano[1], Eithan A. Pozas-Rodríguez[1], María del Rocío Reyero-Saavedra[1†], Alfredo Morales-Salazar[1], Sarah Melissa Lugo-Caro del Castillo[2], Alejandro Sanchez-Flores[3], Verónica Jiménez-Jacinto[3], Jose L. Reyes[4], Damien Formey[2*], and Oswaldo Valdés-López[1*]

[1]Laboratorio de Genómica Funcional de Leguminosas, Facultad de Estudios Superiores Iztacala, Universidad Nacional Autónoma de México, Tlalnepantla, Estado de México, México
[2]Centro de Ciencias Genómicas, Universidad Nacional Autónoma de México, Cuernavaca, Morelos, México
[3]Unidad Universitaria de Secuenciación Masiva y Bioinformática, Instituto de Biotecnología, Universidad Nacional Autónoma de México, Cuernavaca, Morelos, México
[4]Departamento de Biología Molecular de Plantas, Instituto de Biotecnología, Universidad Nacional Autónoma de México, Cuernavaca, Morelos, México

[†]Current Address: Centro de Ciencias Genómicas, Universidad Nacional Autónoma de México, Cuernavaca, Morelos, México

[*]oswaldovaldesl@unam.mx and formey@ccg.unam.mx

Table of Contents:

Supplementary Figure 1. Silencing of *PvAGO5* does not affect root hair development
Supplementary Figure 2. *pPvAGO5:GUS-GFP* activity in rhizobia-inoculated roots and in mature nodules
Supplementary Figure 3. Principal component analysis of the mRNAseq data.
Supplementary Figure 4. Validation of mRNA-seq data by RT-qPCR
Supplementary Figure 5. Transcriptional reprogramming of transporter-encoding genes
Supplementary Figure 6. Transcriptional reprogramming of root nodules symbiosis-related genes in *PvAGO5*-RNAi tissues
Supplementary Figure 7. Transcriptional reprogramming of genes involved in flavonoid biosynthesis

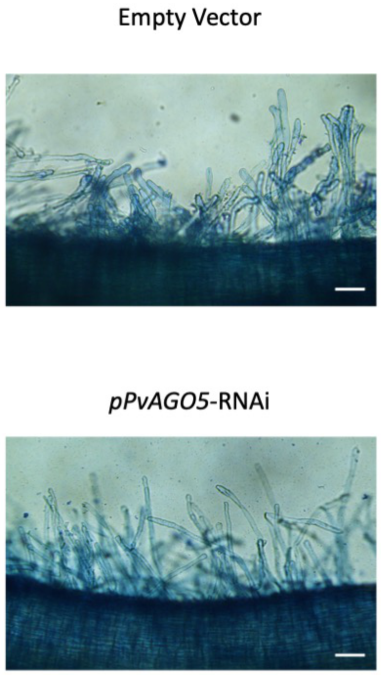

Figure S1. Silencing of *PvAGO5* does not affect root hair development

P. vulgaris composite plants with transgenic roots expressing the *PvAGO5*-RNAi construct were grown under optimal nitrogen conditions for three weeks. Transgenic roots showing TDT fluorescence were collected and observed under a bright-field microscope. Roots were stained with aniline blue to enhance contrast. Scale bars represent 100 μm. Pictures shown are representative of ten biological replicates, each one containing ten transgenic roots.

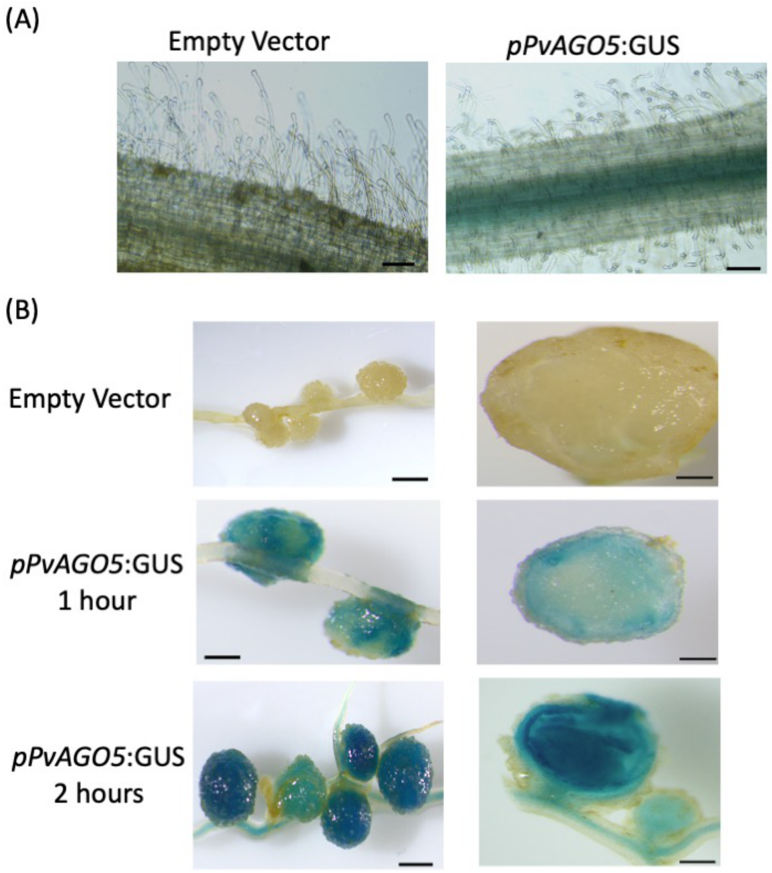

Figure S2. ***pPvAGO5:GUS-GFP* activity in rhizobia-inoculated roots and in mature nodules** *P. vulgaris* composite plants with transgenic roots expressing the *PvAGO5:GUS-GFP* construct were grown under low nitrogen conditions and inoculated with *R. tropici* CIAT899. After one (A) and twenty days (B) of inoculation, transgenic roots showing GFP fluorescence were collected and stained for three for three hours at 37 °C. Scale bars represent 100 μm for the pictures shown in panel A, 1 mm for pictures shown at panel B. Pictures shown are representative of ten biological replicates, each one containing ten transgenic roots.

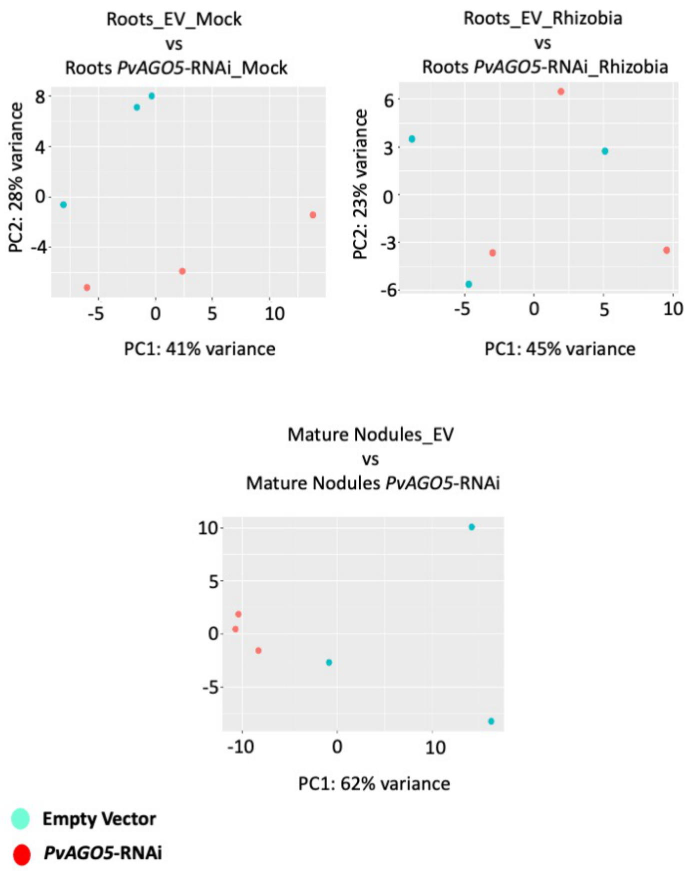

Figure S3. Principal component analysis of the RNAseq

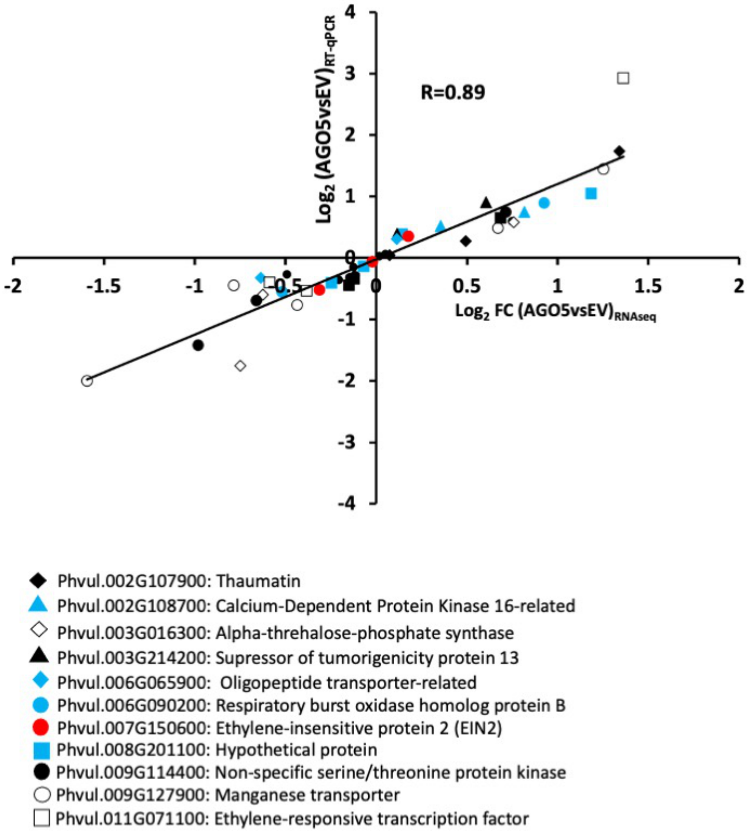

Figure S4. Validation of mRNA-seq data by RT-qPCR

Eleven genes were randomly selected, and their expression was assessed in all the comparisons tested in this study. For this analysis, three biological replicates, each one containing roots, roots bearing nodule primordia, or mature nodules from ten different *P. vulgaris* composite plants, were used. Black line represents lineal trend of the data

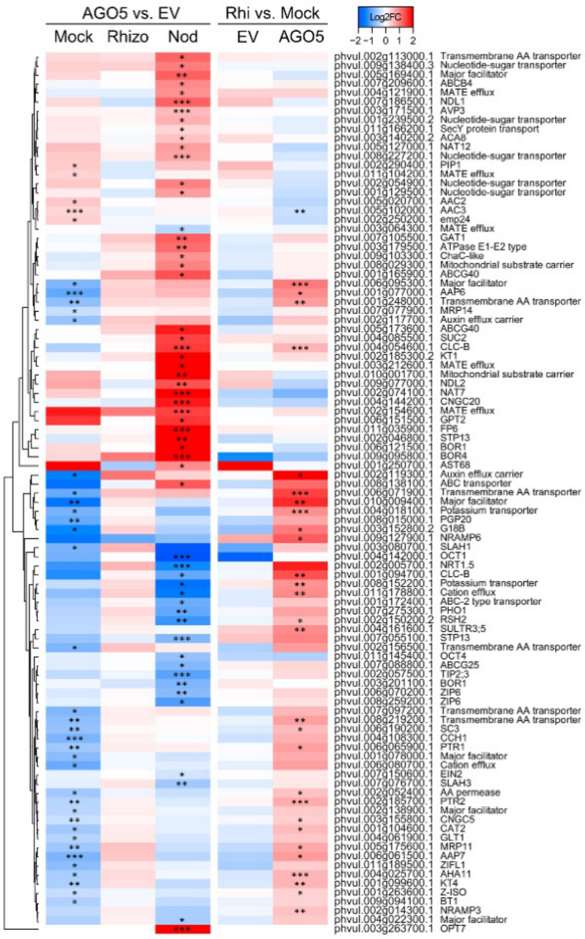

Figure S5. Transcriptional reprogramming of transporter-encoding genes

Heatmap showing Log2 fold-change of transporter-encoding gene transcripts in uninoculated- (Mock) and rhizobia-inoculated roots (rhizobia), as well as in mature nodules (Nod). Genes showing higher or lower expression difference are shown in different shades of red and blue, respectively. Labels at the top of the heatmap located on the left side indicate: Mock= uninoculated transgenic roots; Rhizo= rhizobia-inoculated transgenic roots, and Nod = mature nodules. In all cases the comparison between *PvAGO5*-RNAi vs Empty Vector is shown. Labels at the top of the heatmap located on the right side indicate: EV= transgenic roots expressing the empty vector (control), and *AGO5*: transgenic roots expressing the *PvAGO5-RNAi* construct. In both cases the comparison of rhizobia-inoculated roots vs mock-inoculated roots is shown. Asterisks indicate different levels of statistical significance of the comparison (*: adjusted P-value<0.05; **= adjusted P-value<0.01; ***= adjusted P-value<0.001). Genes with no asterisk are not significantly differentially expressed. Dendrogram represents the transcript clustering based on expression profile.

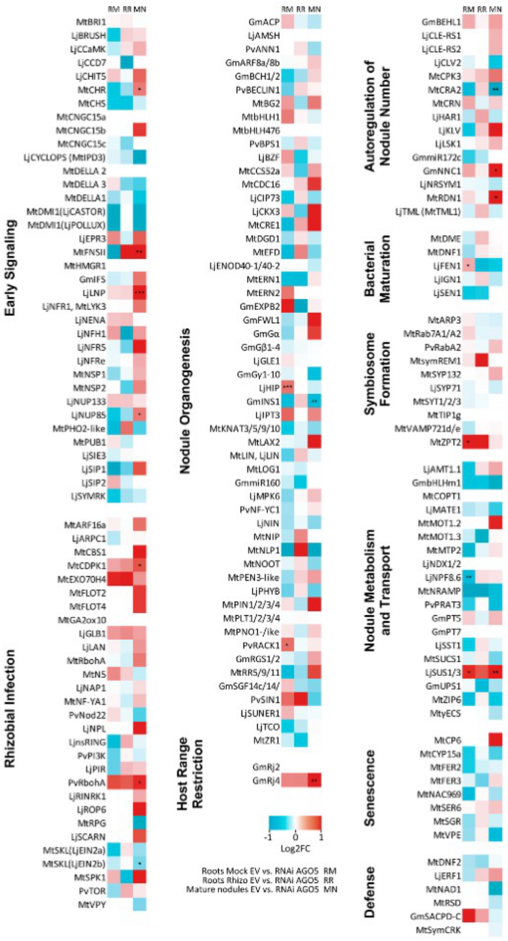

Figure S6. Transcriptional reprogramming of root nodules symbiosis-related genes in *PvAGO5*-RNAi tissues

Heatmap showing Log2 fold-change of gene transcripts involved in different steps of the root nodule symbiosis in uninoculated- (RM) and rhizobia-inoculated roots (RR), as well as in mature nodules (MN). In all cases the comparison between *PvAGO5*-RNAi vs Empty Vector is shown. Genes showing higher expression and lower are shown in different shades of red and blue, respectively. Asterisks indicate different levels of statistical significance of the comparison (*: adjusted P-value<0.05; **= adjusted P-value<0.01; ***= adjusted P-value<0.001). Genes with no asterisk are not significantly differentially expressed. Genes shown were retrieved from (Roy *et al.,* 2020).

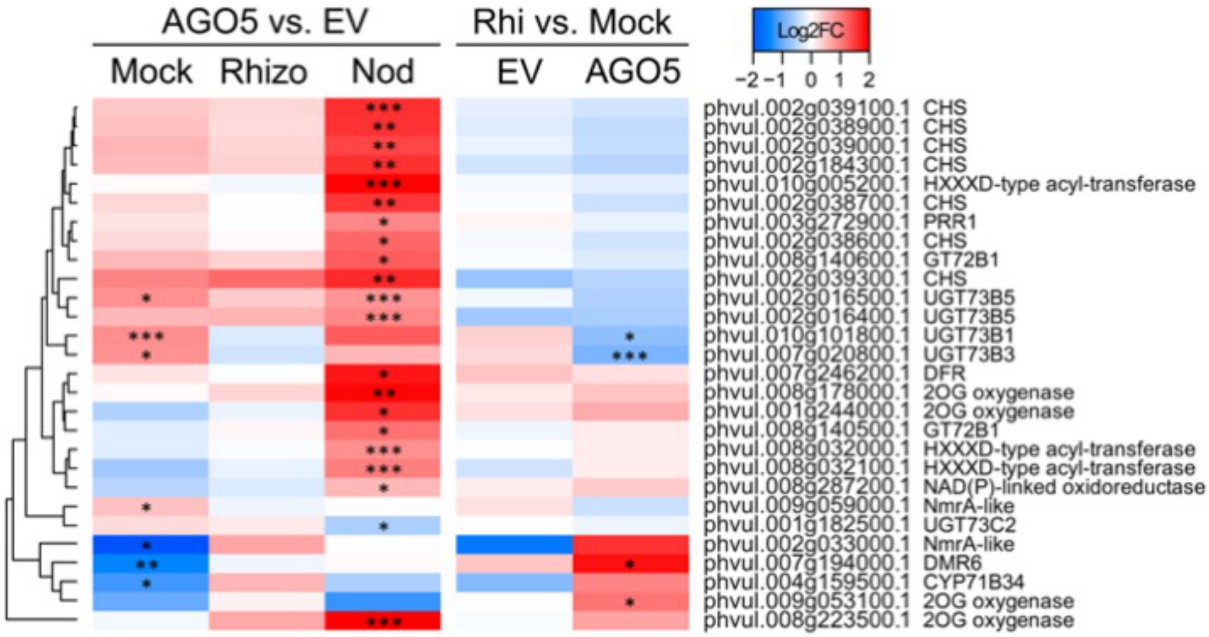

Figure S7. Transcriptional reprogramming of genes involved in flavonoid biosynthesis
Heatmap showing Log2 fold-change of gene transcripts involved in flavonoids biosynthesis in uninoculated- (Mock) and rhizobia-inoculated roots (rhizobia), as well as in mature nodules (Nod). Genes showing higher expression and lower are shown in different shades of red and blue, respectively. Labels at the top of the heatmap located on the left side indicate: Mock= uninoculated transgenic roots; Rhizo= rhizobia-inoculated transgenic roots, and Nod = mature nodules. In all cases the comparison between *PvAGO5*-RNAi vs Empty Vector is shown. Labels at the top of the heatmap located on the right side indicate: EV= transgenic roots expressing the empty vector (control), and *AGO5*: transgenic roots expressing the *PvAGO5-RNAi* construct. In both cases the comparison of rhizobia-inoculated roots vs mock-inoculated roots is shown. Asterisks indicate different levels of statistical significance of the comparison (*: adjusted P-value<0.05; **= adjusted P-value<0.01; ***= adjusted P-value<0.001). Genes with no asterisk are not significantly differentially expressed. Dendrogram (left) represents the transcript clustering based on expression profile.

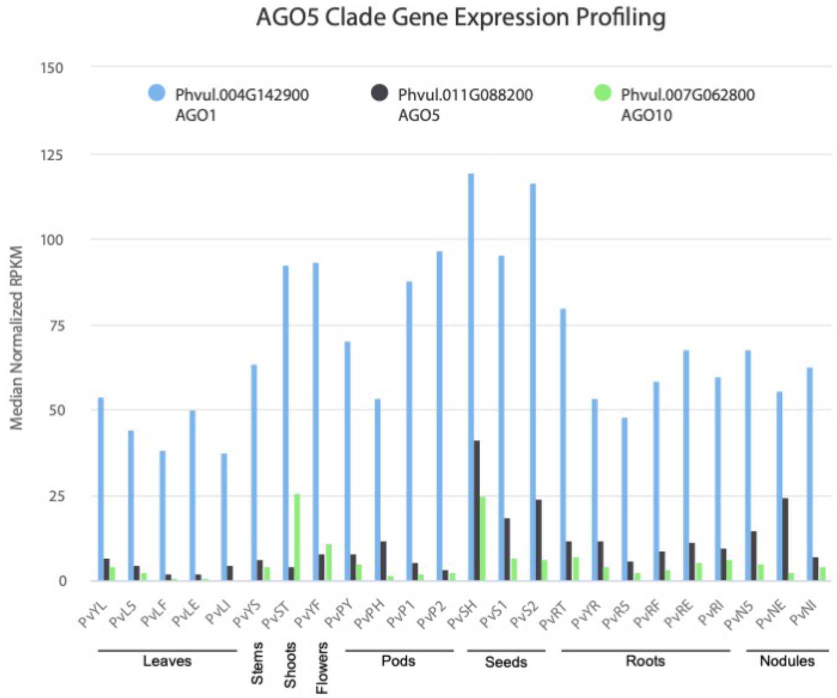

Figure S8. Gene Expression Profiling of AGOs from AGO1/5/10 clade

Expression patterns of samples collected from leave, stems, shoots, flowers, pods, seeds, roots and nodules of *Phaseolus vulgaris* cv. negro jamapa (publicly available at: https://www.zhaolab.org/PvGEA/). Samples were collected at developmentally important time-points spanning symbiosis. Plants were either provided with nutrients via fertilizer or inoculated with either effective or ineffective rhizobium. Abbreviations: PvYL - Fully expanded 2nd trifoliate leaf tissue from plants provided with fertilizer; PvL5 - Leaf tissue collected 5 days after plants were inoculated with effective rhizobium; PvLF - Leaf tissue from fertilized plants collected at the same time of LE and LI; PvLE - Leaf tissue collected 21 days after plants were inoculated with effective rhizobium; PvLI - Leaf tissue collected 21 days after plants were inoculated with ineffective rhizobium; PvYS - All stem internodes above the cotyledon collected at the 2nd trifoliate stage; PvST - Shoot tip, including the apical meristem, collected at the 2nd trifoliate stage; PvFY - Young flowers, collected prior to floral emergence; PvPY - Young pods, collected 1 to 4 days after floral senescence. Samples contain developing embryos at globular stage; PvPH - Pods approximately 9cm long, associated with seeds at heart stage (pod only); PvP1 - Pods between 10 and 11 cm long, associated with stage 1 seeds (pod only); PvP2 - Pods between 12 and 13 cm long associated with stage 2 seeds (pod only); PvSH - Heart stage seeds, between 3 and 4 mm across and approximately 7 mg; PvS1 - Stage 1 seeds, between 6 and 7 mm across and approximately 50 mg; PvS2 - Stage 2 seeds, between 8 and 10 mm across and between 140 and 150 mg; PvRT - Root tips, 0.5 cm of tissue, collected from fertilized plants at 2nd trifoliate stage

of development; PvYR - Whole roots, including root tips, collected at the 2nd trifoliate stage of development; PvR5 - Whole roots separated from 5-day old pre-fixing nodules; PvRF - Whole roots from fertilized plants collected at the same time as RE and RI; PvRE - Whole roots separated from fix+ nodules collected 21 days after inoculation; PvRI - Whole roots separated from fix- nodules collected 21 days after inoculation; PvN5 - Pre-fixing (effective) nodules collected 5 days after inoculation; PvNE - Effectively fixing nodules collected 21 days after inoculation; PvNI - Ineffectively fixing nodules collected 21 days after inoculation. Data available in this browser was previously published in: Jamie A O'Rourke, Luis P Iniguez, Fengli Fu, Bruna Bucciarelli, Susan S Miller, Scott A Jackson, Philip E McClean, Jun Li, Xinbin Dai, Patrick X Zhao, Georgina Hernandez and Carroll P Vance. An RNA-Seq based gene expression atlas of the common bean. BMC Genomics 2014, 15:866.
